# Resistant Secondary Hypertension and Severe Pulmonary Hypertension in a Young Adult due to a Rare Cause

**DOI:** 10.1002/ccr3.71174

**Published:** 2025-10-20

**Authors:** Tingting Su, Yajing Tang, Zhaohui Liu, Lezheng Hou, Xiang Xiao, Yong Yang

**Affiliations:** ^1^ Department of Cardiology The Seventh Affiliated Hospital of Sun Yat‐Sen University Shenzhen Guangdong China; ^2^ Department of Respiration The Seventh Affiliated Hospital of Sun Yat‐Sen University Shenzhen Guangdong China; ^3^ Department of Ultrasonic The Seventh Affiliated Hospital of Sun Yat‐Sen University Shenzhen Guangdong China

**Keywords:** pulmonary hypertension, resistant secondary hypertension, Shone's complex, ventricular dysfunction

## Abstract

Shone's complex (SC), a rare congenital cardiovascular malformation featuring multilevel left‐sided obstructive lesions, presents particular diagnostic challenges in adult populations. The complex interplay between congenital anatomical defects and acquired cardiovascular adaptations in adult SC patients requires multidisciplinary management. Clinicians must maintain a high suspicion for congenital substrates when evaluating young adults with unexplained cardiopulmonary deterioration, particularly when accompanied by refractory hypertension, severe pulmonary hypertension (PH), and right ventricular dysfunction.

**Summary**

- A paradigmatic case of a 28‐year‐old male with refractory secondary hypertension and right ventricular dysfunction secondary to severe pulmonary hypertension ultimately diagnosed with Shone's complex.
- It highlights how congenital anomalies may underlie otherwise unexplained cardiopulmonary deterioration.

## 1 Introduction

Refractory hypertension in young adults necessitates rigorous investigation of secondary etiologies, as precise etiological identification facilitates mechanism‐directed therapies to achieve blood pressure normalization, reverse cardiovascular remodeling, and prevent end‐organ damage from sustained hemodynamic stress [1]. SC, a rare congenital cardiovascular malformation featuring multilevel left‐sided obstructive lesions, presents particular diagnostic challenges in adult populations [2]. Although it predominantly manifests in pediatric populations, advances in congenital heart surgery have resulted in increasing numbers of adult survivors who frequently develop late‐onset complications from persistent left‐sided obstructive lesions. The resultant hemodynamic cascade typically manifests as treatment‐resistant hypertension, progressive PH, and subsequent right ventricular dysfunction—a triad that carries significant morbidity and mortality risks [3]. We present a paradigmatic case of a 28‐year‐old male with refractory secondary hypertension and right ventricular dysfunction secondary to severe pulmonary hypertension, ultimately diagnosed with SC through comprehensive medical history and further examination.

## 2 Case History/Examination

A 28‐year‐old male presented with a 12‐month history of exertional chest oppression, progressive dyspnea (New York Heart Association Class II‐III, NYHA Class II‐III), cephalalgia, and persistent lower limb hypothermia. Physical examination revealed significant interlimb blood pressure discordance: upper extremity blood pressure 190/86 mmHg (right arm) versus lower extremity blood pressure 110/72 mmHg (both legs). Cardiovascular assessment demonstrated the heart was enlarged to the left, a grade 2/6 continuous murmur was audible near the left sternal border at the second intercostal, and a grade 3/6 diastolic rumbling murmur was audible at the apex (P2 > A2). Peripheral vascular evaluation showed diminished femoral artery pulsations bilaterally and non‐palpable dorsalis pedis pulses, accompanied by cutaneous hypoperfusion of the lower extremities. The patient's surgical history was significant for childhood patch aortoplasty for coarctation of the aorta (CoA) repair at age 10. Postoperative surveillance revealed persistent labile hypertension (140–180/80–100 mmHg) refractory to dual antihypertensive therapy (nifedipine sustained‐release 30 mg, once a day + captopril 25 mg three times a day). Notably absent were traditional cardiovascular risk factors, including nicotine exposure, ethanol use, or contributory family history of heritable cardiovascular disorders, and no documented evidence of PH was identified during childhood.

## 3 Differential Diagnosis, Investigations, and Treatment

**FIGURE 1:**
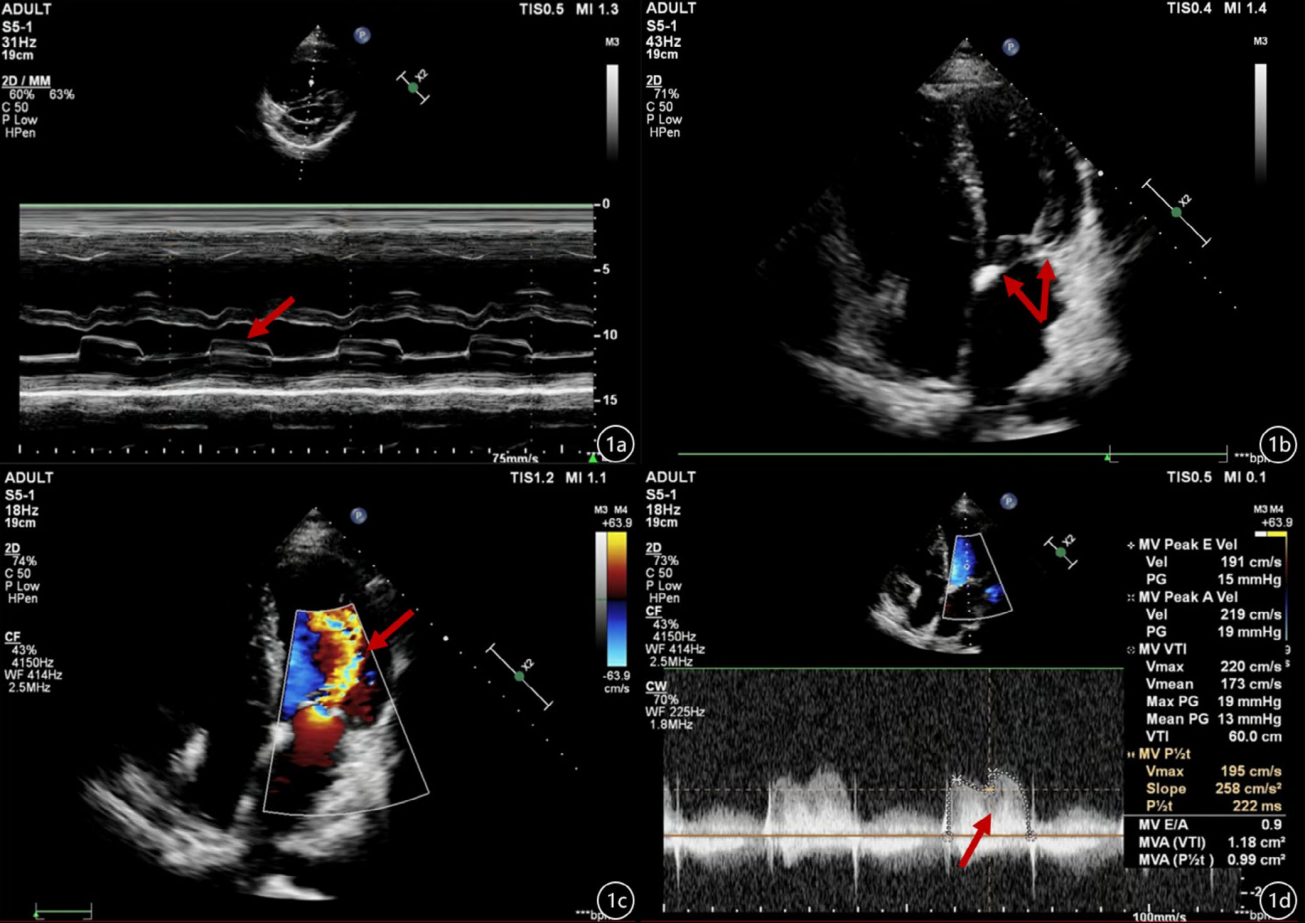
(a) Left ventricular long‐axis view, M‐mode echocardiography showed the wall‐like movement of the mitral valves. (b) Apical four‐chamber view, indicating heart enlargement. The mitral valves were thickened and adhered, with limited movement of the mitral valves on enhanced ultrasound imaging. There was a partial absence of chordae tendineae in both anterior and posterior mitral valve leaflets, and the papillary muscles in the two groups were close together and partially fused. (c) Doppler Color, the effective valve orifice area was reduced, resulting in hemodynamic changes similar to parachute deformity; a multicolored shunt flow signal was seen on mitral regurgitation. (d) The effective orifice area of the stenotic mitral valve was 0.99 cm2 (P1/2t), which resulted in hemodynamic changes similar to those of parachute deformity with a peak diastolic velocity of 2.0 m/s and a peak across mitral valve pressure of 19 mmHg, and a mean gradient of 13 mmHg.

**FIGURE 2:**
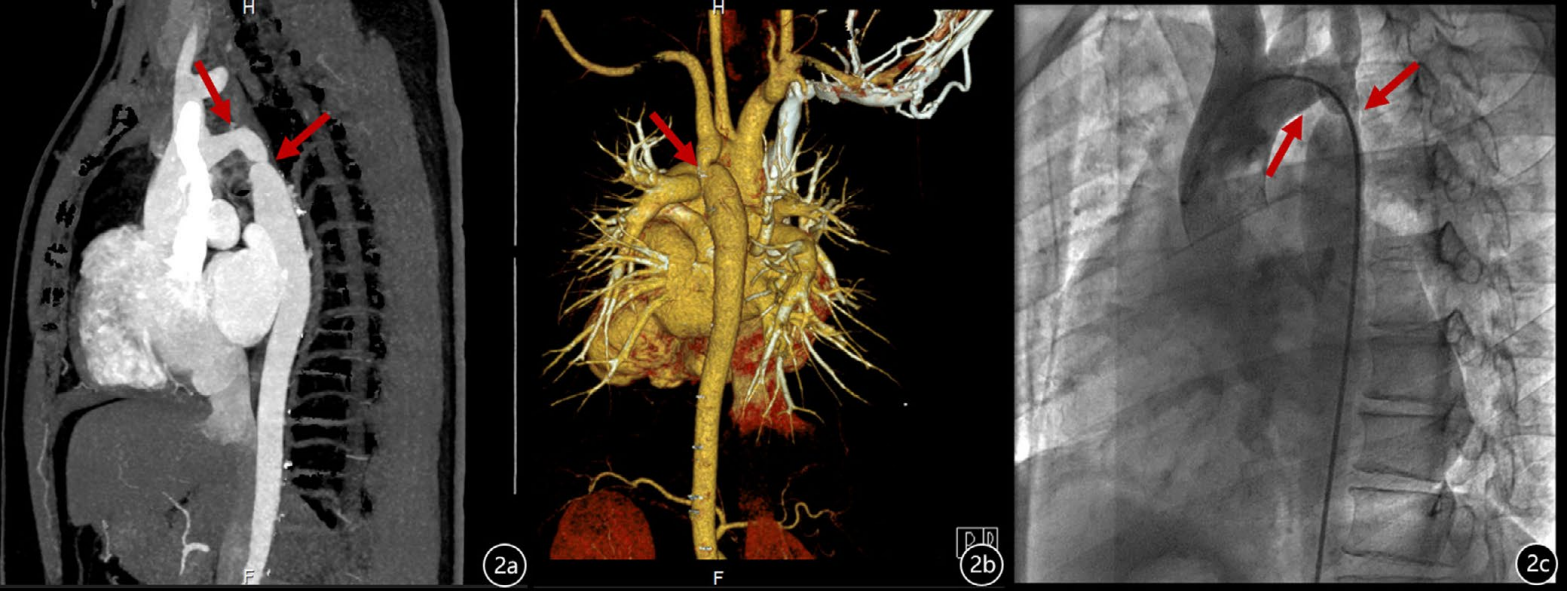
(a, b) Aortic angiography revealed aortic arch and proximal descending aortic stenosis, with a degree of stenosis of approximately 50%–60%. (c) Intraoperative pressure analysis (mmHg) showed that the ascending aortic pressure was 141/78 (99) mmHg, aortic arch pressure was 118/78 (94) mmHg, descending aortic pressure was 106/77 (87) mmHg, and that there was a significant pressure gradient.

Echocardiography confirmed cardiac enlargement. The mitral valves were thickened and adherent, and their movement was limited on enhanced ultrasound imaging. Notably, there was a partial absence of chordae tendineae in both the anterior and posterior mitral valve leaflets. The papillary muscles in the two groups were close together and partially fused, and the effective valve orifice area was reduced; the effective orifice area of the stenotic mitral valve (MVA) was 0.99 cm^2^ (P1/2t), which resulted in hemodynamic changes similar to those of parachute deformity, with a peak diastolic velocity (V_max_) of 2.0 m/s, with the peak trans‐mitral gradient of 19 mmHg, and with the mean gradient of 13 mmHg. M‐mode echocardiography showed the wall‐like movement of mitral valves. Localized narrowing was observed at the aortic arch near the descending aorta; pulmonary artery dilatation was also observed, with a pulmonary artery systolic pressure (PASP) of 87 mmHg (Figure 1). Overall, the echocardiography revealed serious mitral stenosis, CoA, and severe PASP. Computed tomography (CT) confirmed the CoA. It's a pity that RHC was not performed during the current admission owing to the patient's low pain tolerance and subsequent refusal of RHC. To partially mitigate this limitation, we obtained historical hemodynamic data from medical records dated 1 month prior to this hospitalization; these records revealed a pulmonary artery wedge pressure (PAWP) of 60 mmHg. Aortic angiography revealed aortic arch and proximal descending aortic stenosis, with approximately 50%–60% stenosis. Intraoperative pressure analysis (mmHg) showed that the ascending aortic pressure was 141/78 (99) mmHg, aortic arch pressure was 118/78 (94) mmHg, descending aortic pressure was 106/77 (87) mmHg, and that there was a significant pressure gradient (Figure 2).

A clinical diagnosis of CoA with congenital mitral stenosis as a parachute mitral valve (PMV) was made, considered as incomplete SC. We considered his refractory hypertension, severe PH, and right ventricular dysfunction were pathophysiological linked to combined left ventricular inflow and outflow tract obstructions. Cardiovascular surgical consultation has recommended corrective intervention of mitral valve replacement and surgical correction of aortic stenosis. Intraoperative transesophageal echocardiography (TEE) monitoring confirmed the presence of PMV and excluded intracardiac shunts.

## 4 Outcome and Follow‐Up

The patient did well postoperatively with no major complications; the left ventricular inflow tract and outflow tract obstruction were corrected during surgery. Postoperative echocardiograms performed on the first day, at 1 month, and at 3 months after successful mitral valve replacement with relief of mitral stenosis showed that the mitral valve (mechanical) flow spectrum had a hemodynamic V_max_ = 1.2 m/s, along with a significant reduction in PASP = 40 mmHg; nonetheless, PASP did not fully normalize (Table 1). Besides, the patient's blood pressure remains pharmacologically managed with fluctuations in the range of 120–130/60–70 mmHg, consistent with optimal control. Physical examination demonstrates palpable femoral and dorsalis pedis arterial pulses alongside amelioration of lower extremity coldness.

**TABLE 1:** The follow‐up after successful mitral valve replacement and surgical correction of aortic stenosis.

Time	MVA (cm^2^)	V_max_ (m/s)	PG (mmHg)	MG (mmHg)	PASP (mmHg)	BP (mmHg)	NYHA class
Pre‐op	0.99	2.0	19	13	87	140–180/80–100^a^	II‐III
POD1	—	1.2	—	—	40	120–130/60–70^b^	I‐II
POW1	4	1.2	10	4	46	120–140/60–70^b^	I
POM1	4	1.1	9	4	40	120–130/60–70^b^	I
POM3	4	1.1	9	3	38	120–130/60–70^b^	I

Abbreviations: BP, blood pressure; MG, mean trans‐mitral gradient; MVA, mitral valve area; PASP, pulmonary artery systolic pressure; PG, peak trans‐mitral gradient; POD1, postoperative Day 1; POM1, postoperative month 1; POM3, postoperative month3; POW1, postoperative week 1; Pre‐op, pre‐operative; V_max_, the peak mitral diastolic inflow velocity.

^a^ Resistant hypertension.

^b^ Well‐controlled blood pressure.

## 5 Discussion

Arterial hypertension, recognized by the World Health Organization (WHO) as a global health priority, is defined by sustained elevation of systemic blood pressure (≥ 140/90 mmHg) and is pathophysiologically associated with catastrophic multiorgan complications and elevated short‐term mortality. Epidemiologically, approximately 10% of arterial hypertensive cases are classified as secondary hypertension, especially in young populations presenting with severe or refractory hypertension; identifiable secondary etiologies are included but not limited to endocrine (primary aldosteronism, Cushing's syndrome, and pheochromocytoma), renovascular hypertension, or CoA. Precise etiological diagnosis of secondary hypertension is critical, as targeted treatment may achieve blood pressure normalization, reverse cardiovascular remodeling, and prevent end‐organ damage from sustained hemodynamic stress [1]. SC, first described by Shone in 1963, is characterized by four obstructive cardiac anomalies of the systemic circulation: supravalvular mitral membrane, PMV, subaortic stenosis, and CoA [4]. Complete SC includes all four abnormalities, while incomplete SC, more commonly seen, consists of two or three anomalies. Current diagnostic criteria of SC consider it a combination of mitral valve disease coupled with at least one additional left heart obstruction [2]. Owing to the complexity and individual differences in SC, many patients are misdiagnosed or never diagnosed, especially when the left ventricular inflow tract obstruction is mild and clinical symptoms are relatively insidious and nonspecific [5]. Pediatric cohorts predominantly receive isolated CoA‐targeted interventions, yet longitudinal follow‐up reveals adult survivors frequently manifest late‐onset complications arising from persistent left‐sided obstructive lesions [3]. Our patient, initially diagnosed with isolated CoA during childhood and treated with singular CoA surgical correction, progressively developed serious complications of refractory secondary hypertension, right ventricular dysfunction, and severe pulmonary hypertension with age. Comprehensive examinations and clinical features confirmed the diagnosis of incomplete SC, with subsequent cardiothoracic surgical intervention achieving anatomically precise relief of left ventricular inflow and outflow tract obstructions. Postoperative echocardiography data demonstrated significant improvement, and the blood pressure was adequately controlled through a standardized antihypertensive pharmacotherapy regimen.

CoA is frequently associated with concomitant anomalies of other cardiac structures, including mitral stenosis, bicuspid aortic valve, and ventricular septal defect [6]. However, the initial pediatric diagnostic evaluation failed to detect concomitant mitral valve pathology, likely attributable to hemodynamically insignificant left ventricular inflow tract obstruction and the nonspecific clinical presentation during childhood. As the patient aged, mitral valve lesions became symptomatic; severe mitral stenosis and mitral valve pressure resulted in an increase in left atrial pressure and left atrial dilation, leading to a sustained increase in PASP, severe pulmonary hypertension, and finally resulting in right heart dysfunction. If patients with significantly elevated pulmonary artery pressures generally have the worst mitral obstruction and the poorest outcomes. Current consensus guidelines recommend simultaneous intervention for the left ventricular outflow tract obstruction and mitral stenosis (mitral stenosis, MS) when mean gradient in the mitral valve (MV) is ≥ 10 mmHg, or there is PH or there are clinical features of congestive heart failure accompanied by moderate MS. Sonaglioni et al. point out the importance of the incremental diagnostic and prognostic role of exercise stress echocardiography (ESE) in patients affected by mild‐to‐moderate valvulopathies over conventional resting echocardiography, to identify and possibly anticipate the optimal timing for surgical intervention. ESE may be considered for implementation in clinical practice, also for improving the echocardiographic monitoring of SC patients in the future [7]. Once diagnosed, surgery is the mainstay for SC, and the typical surgical strategy is to correct all obstructions [8]. Our patient presented with refractory secondary hypertension, right ventricular dysfunction, and severe pulmonary hypertension; this finally confirmed the diagnosis of incomplete SC. Definitive surgical management entailed anatomical correction of left ventricular inflow and outflow tract obstructions. Finally, the patient underwent successful mitral valve replacement with relief of mitral stenosis, along with a significant reduction in PASP; nonetheless, PASP did not fully normalize. The persistent PH likely reflects underlying pulmonary vascular remodeling secondary to the initial effects of elevated left atrial pressure and pulmonary vascular congestion, with concomitant increases in pulmonary vascular resistance and loss of vascular compliance. The persisting anatomical alterations reverse only slowly in symptomatic patients with severe mitral stenosis despite successful mitral valve replacement [9, 10]. Management of aortic coarctation in SC is vital, as severe narrowing of the aorta can obstruct blood flow. Blood pressure before the obstruction is high, and blood pressure beyond the obstruction is abnormally low, often with weak or absent pulses in the legs, colder extremities, or claudication. A decrease in lower‐body blood pressure in the presence of aortic coarctation may affect the perfusion of lower‐body parts and impair renal blood flow. Further reduction in circulating blood volume may aggravate acute renal failure. The chronic biomechanical stress imposed by both the severity and prolonged duration of CoA drives maladaptive vascular remodeling. Consequently, adult patients with CoA frequently develop refractory hypertension secondary to these structural adaptations [2]. Azarnoosh et al. designed a novel experimental rabbit model of CoA and found vascular alterations, including thickening and stiffening proximal to the coarctation, which worsened with the severity and/or duration of CoA. The wall tension in the proximal region increased markedly with the severity of coarctation. Even mild CoA induced stimuli for remodeling exceed the values observed in adulthood if not treated early [11]. Despite successful anatomical correction achieving post‐repair residual gradients < 20 mmHg, most patients still have persistent blood pressure and require antihypertensive medications, which reflects the irreversible changes in systemic arterial structure and function [12]. Such pathophysiological manifestations were characteristically demonstrated in our patient; although surgical treatment achieved anatomically precise relief of left ventricular outflow tract obstructions, his blood pressure remained high but was adequately controlled through a standardized antihypertensive pharmacotherapy regimen. Thus, elective surgery could be performed to optimize hemodynamics and reduce the risk of aortic dissection, aortic aneurysm rupture, infective endocarditis, and sudden death.

## 6 Conclusion

We present a case of a 28‐year‐old male admitted with refractory secondary hypertension, right ventricular dysfunction, and severe pulmonary hypertension. Comprehensive medical history and further examination confirmed the diagnosis of SC, a rare congenital heart disease characterized by a complex left‐sided cardiac anomaly. This case underscores three critical clinical insights: First, it emphasizes the necessity of systematic investigation for secondary hypertension etiologies in young adults with treatment‐resistant presentations. Second, while SC predominantly manifests in pediatric populations, advances in congenital heart surgery have resulted in increasing numbers of adult survivors who frequently develop late‐onset complications from persistent left‐sided obstructive lesions. These pathophysiological derangements create a cascade of ventricular remodeling, culminating in recalcitrant hypertension, progressive PH, and right heart decompensation. Third, the complex interplay between congenital anatomical defects and acquired cardiovascular adaptations in adult SC patients requires multidisciplinary management. Clinicians must maintain a high suspicion for congenital substrates when evaluating young adults with unexplained cardiopulmonary deterioration, particularly when accompanied by refractory hypertension, severe PH, and right ventricular dysfunction.

## Author Contributions

**Tingting Su:** conceptualization, data curation, methodology, supervision, writing – original draft. **Yajing Tang:** formal analysis, methodology, writing – original draft. **Zhaohui Liu:** data curation, resources. **Lezheng Hou:** data curation, resources. **Xiang Xiao:** supervision, visualization, writing – review and editing. **Yong Yang:** supervision, visualization, writing – review and editing.

## Consent

Written informed consent was obtained from the family member and the patient.

## Conflicts of Interest

The authors declare no conflicts of interest.

## Data Availability

Data available on request from the authors.
